# Chemerin and CMKLR1 expression in human arteries and periadventitial fat: a possible role for local chemerin in atherosclerosis?

**DOI:** 10.1186/1471-2261-14-56

**Published:** 2014-04-30

**Authors:** Christos G Kostopoulos, Sofia G Spiroglou, John N Varakis, Efstratios Apostolakis, Helen H Papadaki

**Affiliations:** 1 Department of Anatomy, School of Medicine, University of Patras, 26500 Rio Patras, Greece; 2 Department of Cardiac Surgery, University Hospital of Ioannina, School of Medicine, University of Ioannina, 45500 Ioannina, Greece

**Keywords:** Periadventitial adipose tissue, Chemerin, CMKLR1, Atherosclerosis, Coronary arteries, Aorta

## Abstract

**Background:**

Depending on their anatomical location, different fat depots have a different capacity to produce bioactive peptides, called adipokines. Adipokines produced by periadventitial fat have been implicated in the pathogenesis of vascular disease, including atherosclerosis. Chemerin is an adipokine with an established role in immunity, adipose tissue function and metabolism, acting in autocrine, paracrine and endocrine manners. We investigated the protein expression of chemerin and its receptor, CMKLR1, in human aortas, coronary vessels and the respective periadventitial adipose tissue and correlated their expression with the presence of atherosclerosis.

**Methods:**

Immunohistochemistry for chemerin and CMKLR1 was performed on human aortic and coronary artery samples including the periadventitial adipose tissue. Aortic and coronary atherosclerotic lesions were assessed using the AHA classification.

**Results:**

Chemerin immunopositivity was noticed in both periadventitial fat depots, in vascular smooth muscle cells and foam cells in atherosclerotic lesions. Periadventitial fat and foam cell chemerin immunopositivity was statistically significantly correlated with the severity of atherosclerosis in both locations. CMKLR1 was expressed in vascular smooth muscle cells and foam cells in aortic and coronary vessels with atherosclerotic lesions. CMKLR1 immunostaining in foam cells was statistically significantly correlated with aortic atherosclerosis.

**Conclusions:**

Our results lend some support to a presumable role of locally produced chemerin in the progression of atherosclerotic lesions, possibly acting through its CMKLR1 receptor. Further research will elucidate the role of chemerin signaling in atherosclerosis.

## Background

Adipose tissue has long been considered as a quiescent organ, offering insulation from the exterior and serving as a passive energy depot [1,2]. However during the past two decades this role has been reviewed, as it was revealed that adipose tissue is an active source of a considerable amount of peptides, called adipokines [1,2]. These substances, acting in an autocrine, paracrine and endocrine manner, have been shown to regulate several body functions and to be implicated in the pathogenesis of clinical entities, such as the metabolic syndrome and atherosclerosis [1,2].

Periadventitial adipose tissue, “tunica adiposa” as proposed [3], constitutes a unique fat depot which is no more considered an innocent bystander, as its role in vascular physiology and pathology through adipokine secretion is now well established [3,4]. Its close vicinity to the vascular wall seems to be of extreme importance, allowing bioactive molecules produced by perivascular fat to easily diffuse into the vascular wall and interact with mural cells, such as vascular smooth muscle cells and endothelial cells, exerting physiological responses [5,6]. In cases where the vascular wall thickness exceeds the limit for passive diffusion, for instance in sites of inflammation and atherosclerotic plaque formation, the hypothesis of the so-called “vasocrine signaling” seems to explain the most possible pathway through which adipokines can reach their targets in deeper vascular wall layers [7]. According to this hypothesis, substances produced near adventitial vasa vasorum can traverse them and be delivered to the intima, so that downstream transport of all adipokines released from periadventitial adipose tissue directly into the arterial wall becomes feasible. In this respect, several adipokines, especially produced by pericoronary epicardial adipose tissue or elsewhere in direct proximity to the vascular wall have been investigated and linked to atherosclerosis, either as promoters or as decelerators of the atherosclerotic process [8-10].

Chemerin is a chemoattractant protein, natural ligand of the G-protein coupled receptor CMKLR1, found in abundancy in human inflammatory fluids [11]. Chemerin has been identified as an adipokine that relates to metabolic syndrome [12-14]. CMKLR1 (also known as ChemR23) is currently the only chemerin receptor (the other two being CCRL2 and GPR1) with clearly described biological actions [14]. Chemerin’s expression in inflamed tissues and its ability for chemotactic recruitment of macrophages and other antigen presenting cells expressing CMKLR1 receptor, as well as the proposed pro- and anti-inflammatory properties of chemerin, suggest a role of this adipokine in inflammatory states and possibly atherosclerosis [14]. Although several aspects of chemerin’s biological functions concerning the cardiovascular system have been investigated [15-17], the contribution of local chemerin expression by perivascular adipose tissue and its interaction with CMKLR1 expressed in the vasculature to the regulation of vascular function is relatively understudied. Therefore, the role of chemerin/CMKLR1 system in vascular inflammation and atherosclerosis is far from clearly elucidated.

We hereby report our results concerning chemerin and CMKLR1 expression in human periaortic abdominal and pericoronary adipose tissue and the adjacent arterial wall. We also report our results concerning associations among the expression of chemerin and its receptor and aortic and coronary atherosclerosis, as well as other clinical parameters.

## Methods

### Tissue specimens

The study has been approved by the Committee on Research and Ethics and the Scientific Committee of the University Hospital of Patras, Greece. Cross-sections of human left coronary arteries (n=40) and abdominal aortas prior to bifurcation into common iliac arteries (n=40), including the periadventitial fat, were obtained from 40 autopsy cases. According to autopsy records, the mean age was 44.5 years (range 14–81 years), the mean Body Mass Index (BMI) was 26.74 kg/m^2^ (range 17.57-32.65 kg/m^2^), 20/40 were hypertensives and 10/40 died of an acute coronary occlusion. The specimens were fixed by immersion in neutrally buffered 10% formalin, dehydrated and routinely processed in paraffin embedding.

### Immunohistochemical staining procedure

Consecutive 4 μm sections of aortic and coronary tissue samples were deparaffinized in xylene and rehydrated in graded alcohols. Endogenous peroxidase activity was quenched by treatment with 1% hydrogen peroxide for 20 min, followed by incubation with appropriate protein blocking solution (3% Bovine Serum Albumin/Tris Buffered Saline). Sections were subsequently incubated overnight in 4°C with rabbit polyclonal primary antibodies (1:1000 anti-Chemerin, Phoenix Pharmaceuticals, Belmont, CA, USA and 1:400 anti-CMKLR1, Abcam, Cambridge, MA, USA). The Envision Plus Detection System kit (DakoCytomation, USA) was used for visualization of bound primary antibody, with 3,3’-diaminobenzidine (DAB) as a chromogen (which yielded brown reaction products). Sections were counterstained with Harris’ hematoxylin solution, dehydrated and mounted. For each antiserum negative controls were performed by replacing the primary antibody with protein blocking solution.

### Histopathological evaluation

The presence of periadventitial adipose tissue and the integrity of the vascular wall were confirmed. Atherosclerotic lesions were assessed according to AHA classification [18] and ranked for statistical reasons in 4 groups: 1: absence of atherosclerotic lesions, 2: AHA type I and II lesions, 3: AHA type III and IV lesions, 4: AHA type Va-c and VI lesions.

### Immunohistological evaluation

All slides were assessed by two pathologists (H.P., J.V.) and two investigators (C.K., S.S.) independently and blinded to the case. Immunoreactivity was graded on a scale of 0–3 according to intensity of cytoplasmic or membrane staining and percentage of immunopositive cells: 0: no staining or <10% positive cells, 1: weak staining in 10-70% positive cells or moderate staining in 10-35% positive cells, 2: weak staining in >70% positive cells or moderate staining in 35-70% positive cells or strong staining in 10-35% positive cells, 3: moderate staining in >70% positive cells or strong staining in >35% positive cells. Microphotographs were obtained using a Nikon DXM 1200C digital camera mounted on a Nikon Eclipse 80i microscope and ACT-1C software (Nikon Instruments Inc., Melville, NY, USA).

### Statistical analysis

Statistical analysis was performed using PASW Statistics 18.0 (SPSS Inc, Chicago, IL, USA). Correlations between expression of proteins, as well as their association with atherosclerosis and age group were evaluated by the Spearman rank-order correlation coefficient. Partial correlation analysis was performed to remove the effect of age and BMI on correlations between protein expression and atherosclerosis. Ordinal data were assessed with non-parametric tests. Significance of protein expression differences between groups with distinct clinical parameters were analysed with Mann–Whitney test. To examine differences in protein expression between depots of the same cases Wilcoxon signed ranks test was used. The significance level was defined as p < 0.05.

## Results

### Evaluation of atherosclerotic lesions

Atherosclerotic lesions ranging from isolated subendothelial macrophages to complicated fibroatheromata were detected in 34/40(85%) aortic tissue and 37/40(92.5%) coronary tissue samples (Tables 1 and 2).

**Table 1:** Expression of chemerin and CMKLR1 in periaortic adipose tissue, aortic VSMCs and atherosclerotic lesion foam cells in correlation with aortic atherosclerosis

		**Aortic atherosclerosis**				
		**NO**	**I-II**	**III-IV**	**V-VI**	
**Total**		**6**	**11**	**4**	**19**	
**IHC score**		**n(%)**	**n(%)**	**n(%)**	**n(%)**	**r and p values**
**Periaortic fat chemerin expression**	**0**	1(2.5)	2(5)	0(0)	0(0)	r=0.824, p < 0.001^a^
	**1**	5(12.5)	5(12.5)	0(0)	0(0)	
	**2**	0(0)	4(10)	1(2.5)	4(10)	
	**3**	0(0)	0(0)	3(7.5)	15(37.5)	
**Aortic VSMC chemerin expression**	**0**	0(0)	0(0)	0(0)	0(0)	r=0.626, p < 0.001^a^
	**1**	3(7.5)	4(10)	1(2.5)	0(0)	
	**2**	3(7.5)	7(17.5)	1(2.5)	9(22.5)	
	**3**	0(0)	0(0)	2(5)	10(25)	
**Aortic foam cell chemerin expression**	**0**	-^b^	7(20.6)	0(0)	0(0)	r=0.670, p < 0.001^a^
	**1**	-	0(0)	0(0)	0(0)	
	**2**	-	1(2.9)	0(0)	1(2.9)	
	**3**	-	3(8.8)	4(11.8)	18(52.9)	
**Aortic VSMC CMKLR1 expression**	**0**	6(15)	8(20)	3(7.5)	13(32.5)	r=0.189, p=0.243
	**1**	0(0)	1(2.5)	1(2.5)	3(7.5)	
	**2**	0(0)	2(5)	0(0)	3(7.5)	
	**3**	0(0)	0(0)	0(0)	0(0)	
**Aortic foam cell CMKLR1 expression**	**0**	-^b^	4(11.8)	0(0)	0(0)	r=0.424, p=0.012^c^
	**1**	-	1(2.9)	2(5.9)	3(8.8)	
	**2**	-	4(11.8)	2(5.9)	7(20.6)	
	**3**	-	2(5.9)	0(0)	9(26.5)	

VSMC=vascular smooth muscle cell, IHC=Immunohistochemistry.

^a^Correlation is significant at the 0.01 level, ^b^Absence of foam cells in case of normal (non atherosclerotic) vessels, ^c^Correlation is significant at the 0.05 level.

**Table 2:** Expression of chemerin and CMKLR1 in pericoronary adipose tissue, coronary VSMCs and atherosclerotic lesion foam cells in correlation with coronary atherosclerosis

		**Coronary atherosclerosis**				
		**NO**	**I-II**	**III-IV**	**V-VI**	
**Total**		**3**	**10**	**9**	**18**	
**IHC score**		**n(%)**	**n(%)**	**n(%)**	**n(%)**	**r and p values**
**Pericoronary fat chemerin expression**	**0**	0(0)	0(0)	0(0)	0(0)	r=0.333, p=0.036^a^
	**1**	1(2.5)	4(10)	2(5)	0(0)	
	**2**	1(2.5)	3(7.5)	3(7.5)	8(20)	
	**3**	1(2.5)	3(7.5)	4(10)	10(25)	
**Coronary VSMC chemerin expression**	**0**	0(0)	0(0)	0(0)	0(0)	r=0.059, p=0.717
	**1**	1(2.5)	1(2.5)	0(0)	1(2.5)	
	**2**	1(2.5)	7(17.5)	7(17.5)	13(32.5)	
	**3**	1(2.5)	2(5)	2(5)	4(10)	
**Coronary foam cell chemerin expression**	**0**	-^b^	2(5.4)	1(2.7)	0(0)	r=0.405, p=0.013^a^
	**1**	-	4(10.8)	3(8.1)	2(5.4)	
	**2**	-	2(5.4)	4(10.8)	10(27)	
	**3**	-	2(5.4)	1(2.7)	6(16.2)	
**Coronary VSMC CMKLR1 expression**	**0**	3(7.5)	6(15)	4(10)	12(30)	r=0.005, p=0.977
	**1**	0(0)	0(0)	3(7.5)	1(2.5)	
	**2**	0(0)	2(5)	2(5)	4(10)	
	**3**	0(0)	2(5)	0(0)	1(2.5)	
**Coronary foam cell CMKLR1 expression**	**0**	-^b^	6(16.2)	3(8.1)	4(10.8)	r=0.317, p=0.056
	**1**	-	2(5.4)	1(2.7)	4(10.8)	
	**2**	-	2(5.4)	3(8.1)	7(18.9)	
	**3**	-	0(0)	2(5.4)	3(8.1)	

VSMC=vascular smooth muscle cell, IHC=Immunohistochemistry.

^a^Correlation is significant at the 0.05 level, ^b^Absence of foam cells in case of normal (non atherosclerotic) vessels.

### Chemerin and CMKLR1 expression in periaortic adipose tissue, aortic vascular smooth muscle cells and foam cells in aortic atherosclerotic lesions

**Figure 1:**
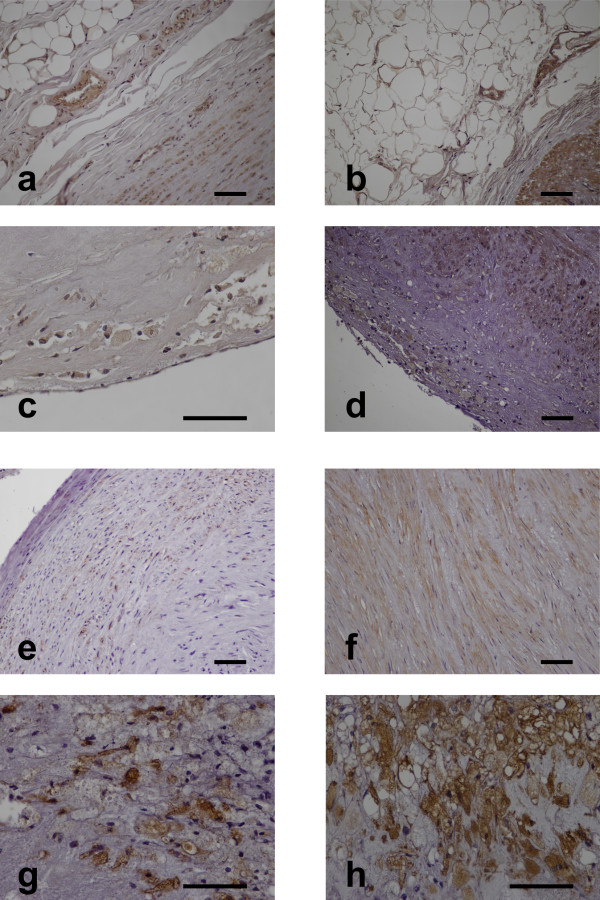
**Chemerin and CMKLR1 expression in periaortic and pericoronary adipose tissue and the adjacent arterial wall (light microscopy, immunohistochemical staining, bar=50 μm). a**. Moderate periaortic adipose tissue and aortic VSMC chemerin immunostaining (x200), **b**. Moderate pericoronary adipose tissue and intense coronary VSMC chemerin immunostaining (x200), **c**. Moderate foam cell chemerin immunostaining in an aortic atherosclerotic lesion (x400), **d**. Intense coronary VSMC and foam cell chemerin immunostaining in a coronary atherosclerotic lesion (x200), **e**. Moderate aortic VSMC CMKLR1 immunostaining (x200), **f**. Moderate coronary VSMC CMKLR1 immunostaining(x200), **g**. Intense foam cell CMKLR1 immunostaining in an aortic atherosclerotic lesion (x400), **h**. Intense foam cell CMKLR1 immunostaining in a coronary atherosclerotic lesion (x400).

Chemerin expression was detected in adipocytes and stromal-vascular cells in 37/40(92.5%) periaortic adipose tissue samples (Table 1, Figure 1a). Chemerin expression was also detected in vascular smooth muscle cells (VSMCs) of the tunica media in 40/40(100%) aortic samples (Figure 1a) and in foam cells of atherosclerotic lesions in 27/34(79.4%) aortic samples (Figure 1c).

CMKLR1 was not expressed in periaortic adipose tissue. CMKLR1 immunoreactivity was detected in VSMCs in 10/40(25%) aortic samples (Figure 1e), while foam cells in aortic atherosclerotic lesions were immunopositive for CMKLR1 in 30/34(88.2%) cases (Figure 1g).

Chemerin expression in periaortic fat was positively correlated with aortic VSMC (r=0.562, p < 0.001) and foam cell chemerin expression (r=0.725, p < 0.001), while aortic VSMC chemerin expression was also positively correlated with aortic foam cell chemerin expression (r=0.493, p=0.003). Another statistically significant positive correlation was noted between chemerin and CMKLR1 expression (r=0.494, p=0.003) in foam cells of aortic atherosclerotic lesions.

### Chemerin and CMKLR1 expression in pericoronary adipose tissue, coronary vascular smooth muscle cells and foam cells in coronary atherosclerotic lesions

Chemerin expression was detected in adipocytes and stromal-vascular cells in 40/40(100%) pericoronary adipose tissue samples (Table 2, Figure 1b). Chemerin expression was also detected in vascular smooth muscle cells (VSMCs) of the tunica media in 40/40(100%) coronary artery samples (Figure 1b,d) and in foam cells of atherosclerotic lesions in 34/37(91.9%) coronary samples (Figure 1d).

CMKLR1 was not expressed in pericoronary adipose tissue. CMKLR1 immunoreactivity was detected in VSMCs in 15/40(37.5%) coronary samples (Figure 1f), while foam cells in coronary atherosclerotic lesions were immunopositive for CMKLR1 in 24/37(64.9%) cases (Figure 1h).

Chemerin expression in pericoronary fat was positively correlated with coronary VSMC (r=0.331, p=0.037) and foam cell chemerin expression (r=0.533, p=0.001). A statistically significant positive correlation was also noted between chemerin and CMKLR1 expression (r=0.336, p=0.042) in foam cells of coronary atherosclerotic lesions.

Interestingly, there were statistically significant differences in protein expression between aortic and coronary samples. In this respect, chemerin and CMKLR1 expression was higher in foam cells of aortic atherosclerotic lesions compared to foam cells of coronary lesions (p=0.024 and p=0.05, respectively), while CMKLR1 expression alone was higher in coronary than in aortic VSMCs (p=0.035).

### Chemerin and CMKLR1 expression in association with atherosclerosis

Several correlations were observed between chemerin and CMKLR1 expression in aortic samples and the severity of aortic atherosclerosis (Table 1). Periaortic fat chemerin expression was positively correlated with atherosclerosis (r=0.824, p < 0.001) and so was aortic VSMC (r=0.626, p < 0.001) and foam cell (r=0.670, p < 0.001) chemerin expression. A positive correlation was also noticed between foam cell CMKLR1 expression and aortic atherosclerosis (r=0.424, p=0.012). Adjusting for age, partial correlations between chemerin expression in periaortic fat (ρ=0.672, p < 0.001), aortic VSMCs (ρ=0.527, p=0.002) and foam cells (ρ=0.610, p < 0.001) and aortic atherosclerosis were still present. When controlling for BMI, partial correlations between periaortic fat chemerin (ρ=0.793, p < 0.001), aortic VSMC chemerin (ρ=0.587, p < 0.001), aortic foam cell chemerin (ρ=0.711, p < 0.001) and aortic foam cell CMKLR1 (ρ=0.434, p=0.012) expression and aortic atherosclerosis remained strong.

Coronary atherosclerosis was also correlated with chemerin and CMKLR1 expression in coronary samples (Table 2). Pericoronary fat chemerin expression was positively correlated with coronary atherosclerosis (r=0.333, p=0.036) and so was coronary foam cell chemerin expression (r=0.405, p=0.013). These correlations remained strong when adjusting for age (ρ=0.407, p=0.014 and ρ=0.377,p=0.023, respectively) and BMI (ρ=0.385, p=0.02 and ρ=0.410, p=0.013, respectively). A marginally non significant correlation was observed between coronary foam cell CMKLR1 expression and coronary atherosclerosis (r=0.317, p=0.056) and remained non significant when controlling for age (ρ=0.290, p=0.086) and BMI (ρ=0,328, p=0.051).

Interestingly, coronary atherosclerosis was positively correlated with periaortic fat and aortic VSMC chemerin expression (r=0.438, p=0.005 and r=0.406, p=0.009, respectively).

### Expression of chemerin and CMKLR1 in association with age and clinical parameters

Dividing the cases in age groups with 20-year intervals, statistically significant correlations were noticed between protein expression and age groups (Table 3). Periaortic fat chemerin expression was increasing with age (r=0.664, p < 0.001) and so was aortic VSMC chemerin expression (r=0.430, p=0.006). Chemerin and CMKLR1 expression in foam cells of aortic atherosclerotic lesions was also increasing with age (r=0.554, p=0.001 and r=0.433, p=0.01, respectively). No correlations were observed between protein expression and age group in the case of coronary samples.

**Table 3:** Significant correlations between expressions of chemerin and CMKLR1 and age group

		**Age group**					
		**0-20**	**21-40**	**41-60**	**61-80**	**80+**	
**IHC score**		**n(%)**	**n(%)**	**n(%)**	**n(%)**	**n(%)**	**r and p values**
Total		5(12.5)	13(32.5)	12(30)	9(22.5)	1(2.5)	
**Periaortic fat chemerin expression**	**0**	2(5)	1(2.5)	0(0)	0(0)	0(0)	r=0.664, p < 0.001^a^
	**1**	2(5)	7(17.5)	1(2.5)	0(0)	0(0)	
	**2**	1(2.5)	1(2.5)	6(15)	1(2.5)	0(0)	
	**3**	0(0)	4(10)	5(12.5)	8(20)	1(2.5)	
Total		5(12.5)	13(32.5)	12(30)	9(22.5)	1(2.5)	
**Aortic VSMC chemerin expression**	**0**	0(0)	0(0)	0(0)	0(0)	0(0)	r=0.430, p=0.006^a^
	**1**	1(2.5)	6(15)	1(2.5)	0(0)	0(0)	
	**2**	4(10)	5(12.5)	5(12.5)	6(15)	0(0)	
	**3**	0(0)	2(5)	6(15)	3(7.5)	1(2.5)	
Total		3(8.8)	9(26.4)	12(35.3)	9(26.4)	1(2.9)	
**Aortic foam cell chemerin expression**^**b**^	**0**	2(5.9)	4(11.8)	1(2.9)	0(0)	0(0)	r=0.554, p=0.001^a^
	**1**	0(0)	0(0)	0(0)	0(0)	0(0)	
	**2**	0(0)	1(2.9)	1(2.9)	0(0)	0(0)	
	**3**	1(2.9)	4(11.8)	10(29.4)	9(26.5)	1(2.9)	
Total		3(8.8)	9(26.4)	12(35.3)	9(26.4)	1(2.9)	
**Aortic foam cell CMKLR1 expression**^**b**^	**0**	2(5.9)	2(5.9)	0(0)	0(0)	0(0)	r=0.433, p=0.01^c^
	**1**	0(0)	1(2.9)	4(11.8)	1(2.9)	0(0)	
	**2**	0(0)	5(14.7)	5(14.7)	3(8.8)	0(0)	
	**3**	1(2.9)	1(2.9)	3(8.8)	5(14.7)	1(2.9)	

VSMC=vascular smooth muscle cell, IHC=Immunohistochemistry.

^a^Correlation is significant at the 0.01 level, ^b^ Foam cells present at n=34 lesions, ^c^Correlation is significant at the 0.05 level.

Clinical parameters, namely hypertension and acute coronary occlusion were also associated with chemerin expression (Table 4). Statistically significant higher expression of chemerin (p=0.044) in periaortic fat was observed in cases with hypertension, compared with normotensive cases. Interestingly, increased chemerin expression in VSMCs of aortic media was also noted in hypertensives (p=0.021). Finally, significantly increased foam cell chemerin expression in coronary lesions was detected in cases with acute coronary occlusion (p=0.018).

**Table 4:** Significant differences in the expression of chemerin in hypertensives and in case of acute coronary occlusion as a cause of death

**Total**		**No hypertension**	**Hypertension**	
		**20**	**20**	
	**IHC score**	**n(%)**	**n(%)**	**p value**
**Periaortic fat chemerin expression**	**0**	3(7.5)	0(0)	p=0.044^a^
	**1**	7(17.5)	3(7.5)	
	**2**	3(7.5)	6(15)	
	**3**	7(17.5)	11(27.5)	
**Aortic VSMC chemerin expression**	**0**	0(0)	0(0)	p=0.021^a^
	**1**	5(12.5)	3(7.5)	
	**2**	13(32.5)	7(17.5)	
	**3**	2(5)	10(25)	
		**Other cause**	**Acute coronary occlusion**	
**Total**		**30**	**10**	
	**IHC score**	**n(%)**	**n(%)**	**p value**
Total		27(72.9)	10(27)	
**Coronary foam cell chemerin expression**^**b**^	**0**	3(8.1)	0(0)	p=0.018^a^
	**1**	8(21.6)	1(2.7)	
	**2**	12(32.4)	4(10.8)	
	**3**	4(10.8)	5(13.5)	

VSMC=vascular smooth muscle cell, IHC=Immunohistochemistry.

^a^Difference is significant at the 0.05 level, ^b^Foam cells present at n=37 lesions.

## Discussion

Since our previous report of chemerin – among other adipokines – expression in periadventitial adipose tissue and its correlation with atherosclerosis [19], a growing amount of evidence about chemerin and its possible role in vascular inflammation and atherosclerosis have come to our knowledge [15-17,20]. However, the exact mechanisms of action and the role of chemerin and its receptors yet remain unclarified, as remains the contribution of locally produced chemerin and its receptors within atherosclerotic vessels. Hence, in this study we sought to find the existing, if any, correlations between chemerin and CMKLR1 local expression in periadventitial adipose tissue and the related vascular wall and the extent of atherosclerotic lesions in aortic and coronary tissue samples.

We found chemerin to be expressed in adipocytes and perivascular adipose tissue stromal-vascular cells, as well as vascular smooth muscle cells (VSMCs) in the aortic and coronary arterial wall. Moreover, significant chemerin immunostaining was observed among foam cells in atherosclerotic lesions. Chemerin has been previously reported to be expressed by these cell types [12-14,16,19], thus our findings merely confirmed this observation. CMKLR1 expression by foam cells in atherosclerotic lesions is an expected finding as its expression by macrophages is well known [11,21]. VSMC expression of CMKLR1 was also not surprising as it has been previously been reported to take part in VSMC phenotype modulation and has been linked to peripheral atherosclerosis [22]. In our material, CMKLR1 was not found to be expressed by mature adipocytes, such as those present in periadventitial adipose tissue. At this point it is of some interest to note that neither chemerin nor CMKLR1 showed any significant endothelial immunostaining in our study, unlike other investigators’ reports [13,23]. However, taken altogether, these findings reveal a significant presence of chemerin/CMKLR1 signaling axis in the inflammatory atherosclerotic milieu.

The expression pattern of chemerin throughout the vascular wall in both sites in our study suggests that a possible coordination mechanism may exist, as all cell types seem to contribute proportionally in chemerin production. In explanation, both in aortas and in coronary arteries there is a strong correlation among chemerin expression in perivascular adipocytes, VSMCs and foam cells. Along with the fact that chemerin expression in foam cells seems to be correlated with their CMKLR1 expression, it is quite clear that there might be a role for chemerin as a regulator of macrophage action in sites of vascular inflammation. This observation comes in concordance with a previous investigation [21] reporting chemerin’s ability to promote macrophage adhesion to extracellular matrix proteins and adhesion molecules through CMKLR1 signaling and suggesting a novel role for chemerin in the recruitment and retention of macrophages at sites of inflammation.

Several correlations between chemerin/CMKLR1 expression in our material and atherosclerosis were noted. Atherosclerosis severity was positively correlated with perivascular adipocyte and foam cell chemerin expression in both sites under investigation, namely aortas and coronary arteries. VSMC chemerin was positively correlated with atherosclerosis only in aortic lesions, while foam cell CMKLR1 expression was strongly positively correlated with aortic atherosclerosis, but only marginally with coronary atherosclerosis. Moreover, most correlations remained strong even after adjusting for age and BMI. Overall, our findings suggest a role for local chemerin production in the atherosclerotic process, as previously described [19], involving its CMKLR1 receptor. However, this role may be subject to interactions with other cellular components within the vascular wall, as well as other receptors and ligands [14,22,24], rendering the full understanding of chemerin/CMKLR1 axis function even more complex. For instance, resolvin E1 is an endogenous lipid mediator found to mediate resolution of inflammation through leucotriene B4 receptor BLT1 and CMKLR1 activation [22,24], exerting anti-inflammatory actions via the same receptor as chemerin. Thus, CMKLR1 may possibly act as a receptor for both pro-inflammatory and anti-inflammatory signals in diseased vessels. In addition, CMKLR1 presence in VSMCs seems to promote the quiescent, contractile phenotype when activated by resolvin E1 [22], however little is known about chemerin-induced CMKLR1 activation in VSMCs, which may promote an opposite phenotypic switch, resulting in migration, proliferation and progression of atherosclerotic plaques. Finally, the effects of chemerin itself on macrophages/foam cells and endothelial cells is far from elucidated, as conflicting evidence about its possible pro- or anti-inflammatory and atherogenic role have been emerging [14,21,23,25-27]. Nevertheless, co-localization of chemerin and CMKLR1 in large amounts in foam cells of severe atherosclerotic lesions suggests a putative role for this signaling axis in macrophage recruitment and foam cell formation in human atherosclerotic lesions.

Interestingly, high serum concentrations of chemerin in several studies appear consistently associated with human vascular inflammation and atherosclerosis, especially coronary artery disease [15,17,28]. In this respect, our observation that coronary atherosclerosis severity was positively correlated with periaortic adipose tissue and aortic VSMC chemerin expression might reflect the significant contribution of visceral fat (a special compartment of which is abdominal perivascular adipose tissue) in circulating chemerin levels, which in turn activate the constitutively expressed CMKLR1 in macrophages and possibly other vascular cell types, leading in acceleration of the atherosclerotic process.

It may be of some interest to note the positive correlation of periaortic fat, aortic VSMC and aortic foam cell chemerin expression, as well as aortic foam cell CMKLR1 expression with age, whereas there was no such correlation in the instance of coronary samples. This finding, which is in agreement with previous reports regarding serum chemerin levels [13], may suggest an effect of senescence on chemerin expression, although in a different manner in separate locations, but this is yet to be confirmed by future investigation.

In the present study, we also demonstrated an increased chemerin expression in periaortic adipose tissue and aortic VSMCs in hypertensive cases rather than in normotensive. There have been reports, since the identification of chemerin as an adipokine, about its close association with the metabolic syndrome and related co-morbidities [13,15,20,29]. As a basic constituent of the metabolic syndrome, arterial hypertension has been linked to high serum chemerin levels in most of these studies [13,20,30]. Thus, our results agree with a potential role for chemerin in blood pressure regulation, in the context of metabolic syndrome.

A last, but of some importance, finding in this study was a correlation between foam cell chemerin in coronary lesions with acute coronary occlusion as a cause of death in our cases. As already discussed, whether high serum chemerin levels promote atherosclerosis, especially coronary artery disease, is still in debate [30], with growing recent data from human studies confirming this theory [15,28]. Moreover, although new research results support a possible protective role for chemerin/CMKLR1 [26], there is a large amount of evidence sustaining a pro-inflammatory role for chemerin [17,21,23,25]. To be more specific, there is a well established role for chemerin in CMKLR1-positive macrophage recruitment and retention in sites of inflammation, through enhanced adhesion to extracellular matrix and adhesion molecules [21], while a potential angiogenic and gelatinolytic (through MMP-2 and MMP-9 induction) influence on endothelial cells has been reported for chemerin [23]. Chemerin has also been demonstrated to affect adhesion molecule (ICAM, E-selectin) expression by endothelial cells [17], while a supposed direct anti-inflammatory role of chemerin through CMKLR1 on human macrophages was ruled out in a recent ex-vivo study [25]. Moreover, recent findings in an animal model suggest that chemerin reduces nitric oxide (NO) production, enhances NO breakdown and also decreases NO-dependent cGMP signalling, thereby inducing vascular dysfunction [31]. Taken altogether, these facts support a potential role for chemerin in atherosclerotic plaque progression and destabilization. Acting via CMKLR1 activation, but not exclusively, chemerin may contribute in lipid core enlargement, fibrous cap thinning and erosion, thus leading in plaque rupture and acute coronary events, in case of coronary lesions.

## Conclusions

In this study we demonstrate the expression of chemerin and its CMKLR1 receptor in human aortas and coronary arteries with varied severity of atherosclerotic lesions in situ. The observed correlations between protein expression and atherosclerosis lend some support to the hypothesis that local chemerin expression within the diseased vascular wall, acting through its CMKLR1 receptor may trigger complex cell-cell interactions, contributing – most probably – in atheromatous plaque progression. However, ruling out the effects of other CMKLR1 ligands, such as lipid mediator resolvin E1, as well as other chemerin receptors, such as CCRL2, seems to be the challenge, so as to better understand the chemerin/CMKLR1 signaling axis. Extended investigation, integrating ex vivo experimental methodology, in vivo research on animal models of atherosclerosis (expressing chemerin/CMKLR1 and silenced for one or both of these proteins) and – ideally – translational research in humans would clarify the role of this regulating system. Chemerin and CMKLR1, as already discussed, seem to play a pivotal role in vascular inflammation and atherogenesis. Local chemerin production by several components of the vascular wall may be of distinguishing importance, due to the proximity to lesions and may also have varying effects, depending on local interactions. Further research will elucidate the role of chemerin signaling in atherosclerosis.

## Competing interests

The authors declare that they have no competing interests.

## Authors’ contributions

CK and SS conceived of the study, participated in its design and coordination, conducted experiments, assessed the slides, performed the statistical analysis and drafted the manuscript. JV and HP participated in the design and coordination of the study, assessed the slides, contributed in the interpretation of the results and revised the manuscript. EA contributed in the design and coordination of the study, as well as the interpretation of the results and revision of the manuscript. All authors read and approved the final manuscript. All authors have substantially contributed to the present work.

## Pre-publication history

The pre-publication history for this paper can be accessed here:

http://www.biomedcentral.com/1471-2261/14/56/prepub
